# *Cryptosporidium* Infection Increases the Risk for Chronic Diarrhea Among People Living With HIV in Southeast Asia: A Systematic Review and Meta-Analysis

**DOI:** 10.1177/1010539519895422

**Published:** 2020-02-10

**Authors:** Wiwien S. Utami, Elsa H. Murhandarwati, Wayan T. Artama, Hari Kusnanto

**Affiliations:** 1 Jember University, Jember, Indonesia; 2 Universitas Gadjah Mada, Yogyakarta, Indonesia

**Keywords:** systematic review, meta-analysis, *Cryptosporidium*, cryptosporidiosis, chronic diarrhea, people living with HIV, Southeast Asia

## Abstract

We conducted a systematic review research and meta-analysis to reveal the relationship between the risk of chronic diarrhea and *Cryptosporidium* infection in people living with HIV in Southeast Asia. We performed online peer-reviewed literature research from January 2005 to December 2017, which included PubMed, Science Direct, ProQuest, EBSCO, Cochrane, and Web of Science databases. Calculation of size effects in the meta-analysis was performed by STATA 13.0 software to estimate relative risks (RRs) with 95% confidence intervals (CIs) for any associations. Seven cross-sectional research articles were recruited in this study based on the inclusion and exclusion criteria. Our analysis revealed a significant relationship between cryptosporidiosis and the risk of chronic diarrhea in people living with HIV, with RR = 1.325; 95% CI = 1.157 to 1.517; and *P* < .000. Our results suggested that cryptosporidiosis increases the risk of chronic diarrhea, and low CD4^+^ lymphocyte cell counts aggravate the degree of diarrhea. Therefore, clinicians should be more aware in treating HIV-positive people, especially those with low CD4^+^ cell counts, and we suggest that *Cryptosporidium* laboratory examinations be conducted immediately.

## What We Already Know

- Southeast Asia is facing a severe problem with HIV/AIDS due to its high prevalence and rapid spread. *Cryptosporidium* infection was reported as a causative agent of diarrhea among immunocompromised patients.
- Cryptosporidiosis remains a common cause of chronic diarrhea in people living with HIV. It is responsible for most deaths in children younger than 5 years in developing countries, with up to 74% of diarrheal stools demonstrating the organism.
- Diarrhea can persist for several months in patients with CD4^+^ T-lymphocyte counts less than 50 to 100 cells/mm^3^, resulting in severe dehydration, weight loss, malnutrition, prolonged hospitalization, and even death.

## What This Article Adds

- Our findings proved that cryptosporidiosis in people living with HIV can aggravate the degree of diarrhea. Our study found that additional laboratory tests for *Cryptosporidium* infection have been rarely performed in people living with HIV. Based on the result, it is recommended for practitioners in the clinical examination to use clinical and paraclinical characteristics in making the diagnosis of HIV/AIDS and other opportunistic parasitic diseases.
- The recommendation is important to prevent persistent and life-threatening diarrhea; therefore, cryptosporidiosis as the cause of the diarrhea must be treated first.

## Introduction

Cryptosporidiosis is a disease caused by a microscopic parasite, *Cryptosporidium* species,^1^ an intracellular obligate protozoan that infects microvilli epithelial cells in the digestive tract.^2,3^ Diarrhea is one of the leading causes of mortality that is responsible for more than 1 to 6 million deaths worldwide in 2016.^4^ *Cryptosporidium* is one of the three etiologies responsible for most deaths in children younger than 5 years^5^ and will be more severe if it occurs in children living with HIV.^6^ Transmission is efficient and only requires few dozen oocysts to cause disease in healthy individuals and can become severe in immunocompromised individuals.^7,8^ Currently, cryptosporidiosis is a significant cause of morbidity and mortality worldwide, and it is the leading cause of chronic diarrhea in HIV-positive people.^9-11^ Chronic diarrhea often becomes a significant burden for people living with HIV (PLHIV), especially in developing countries.^12^ Patients with low CD4^+^ T-lymphocyte counts and antiretroviral therapy (ART)-naïve patients had higher prevalence to be infested by *Cryptosporidium* than other patients (*P* < .01). Diarrhea can persist for several months in patients with CD4^+^ T-lymphocyte counts less than 50 to 100 cells/mm^3^, resulting in severe dehydration, weight loss, malnutrition, extended hospitalization, and even death.^13^

Cryptosporidiosis is a self-limiting disease in the immunocompetent but not in the immunocompromised patient, where it can be life-threatening. Invasive *Cryptosporidium* infection of the small intestine damages the intestinal epithelium and disrupts absorption and barrier function of intestine,^14^ leading to mild-to-severe diarrhea.^5^ The treatment is basically to reduce the duration of diarrhea, prevent complications, and eliminate the organism from the host, in order to reduce comorbidity and mortality. Research proves that the treatment of diarrhea in PLHIV is not effective enough if there is a *Cryptosporidium* infection.^15^ Diarrhea still occurs despite paromomycin administration. It shows that diarrhea treatment alone is not adequate in PLHIV, but cryptosporidiosis as the cause of the diarrhea must be treated first. Effective treatment of cryptosporidiosis will be useful as an adjuvant to ART, as well as in settings where antiretrovirals are either too expensive or not available, for example, for malnourished children in the developing world. Also, if effective treatments were available, cancer and posttransplant patients would not be required to interrupt immunosuppression in order to treat cryptosporidiosis.^16^

Chronic diarrhea that is persistent due to *Cryptosporidium* infection in PLHIV can be potentially life-threatening, and it is known as a cause of poor absorption of antiviral drugs and treatment failure in HIV infection.^10^ HIV-seropositive patients with CD4^+^ ≤50 cell/mm^3^ usually have severe clinical symptoms, including diarrhea. Typically, patients with CD4^+^ ≤200 cell/mm^3^ have increased susceptibility to *Cryptosporidium* infection. Diarrhea is a major concern for HIV-seropositive patients because it can lower their quality of life and causes severe pain, and even death. This continuous diarrhea causes about 40% of deaths in PLHIV in Kenya.^17^ Diarrhea is closely related to low CD4^+^ counts and reported as the second most frequent cause of hospital visits in several developing countries. Diarrhea that becomes profuse is usually followed by significant weight loss, anorexia, malabsorption syndrome, and fever, and accompanied by abdominal pain.^18^

Annually, approximately 8500 cases of cryptosporidiosis are reported in the United States^19^; while Brazil and Africa reported that the prevalence of cryptosporidiosis was 3.5% to 22.4% and around 50% from PLHIV with a low CD4^+^ cells, respectively.^20-22^ The incidence of *Cryptosporidium* infections was found to be 10.1% of PLHIV in China,^23,24^ 7.6% cases of cryptosporidiosis were reported to be HIV-seropositive patients in Iran,^25^ while 71.4% of that prevalence were associated with diarrhea. Approximately 28.6% of cases of *Cryptosporidium* infection in India^26^ and 4.3% of cases in Bangladesh were asymptomatic.^27-29^ In Malaysia, 12.4% of PLHIV were infected with *Cryptosporidium.*^30^ In Cambodia, in 2006, the prevalence of cryptosporidiosis in PLHIV was 40% and 53%, in the symptomatic and asymptomatic groups, respectively, indicating underdiagnosis of *Cryptosporidium* infection.^31^ In Indonesia, a total of 4.9% of PLHIV were reported to be positively infected by cryptosporidiosis and/or *Blastocystis hominis* in 2009.^18^ In 2013, 77.7% of HIV-seropositive patients were reported infected by *Cryptosporidium hominis*, and a total of 5.5% of patients were affected by sev-eral *Cryptosporidium* spp (*Cryptosporidium hominis, Cryptosporidium meleagridis, Cryptosporidium felis*, and *Cryptosporidium parvum*).^15^

In Southeast Asia, *Cryptosporidium* infections were reported from several studies conducted in Cambodia, Indonesia, Lao People’s Democratic Republic, Malaysia, Myanmar, Philippines, Singapore, Thailand, and Vietnam.^32,33^ Many factors cause transmission of infectious diseases including population movements between neighboring countries, rapid modernization, economic and political development, and the increasing of population growth.^34^ These factors, together with the increasing of AIDS cases in tropical and subtropical countries, are very conducive to the proliferation of many opportunistic agents of infection.^35^ Furthermore, due to substantial changes in population growth and appropriate climate conditions, Southeast Asia is a hot spot for the emergence of new infectious diseases.^33^ Southeast Asia is recognized as an “epicenter” for emerging infectious diseases^36^ due to its tropical or subtropical climate that is conducive to the propagation of many protists, including cryptosporidiosis.^37^ Currently, a number of Southeast Asian countries face a severe yet likely underestimated problem with HIV/AIDS, due to its high prevalence and rapid spread for economic and political reasons.^32^ This situation emphasizes the need to be aware of *Cryptosporidium* infection in PLHIV due to the risk of becoming chronic or persistent diarrhea. This study aimed to prove the risk of chronic diarrhea due to *Cryptosporidium* infection in PLHIV in the Southeast Asian region using a systematic review and meta-analysis.

## Methods

This research was a quantitative study with meta-analysis study design. Meta-analysis is an analysis of several studies using a systematic approach and statistical techniques to identify, assess, and combine the results of relevant research to reach a stronger conclusion. This meta-analysis was conducted using the Meta-analysis of Observational Studies in Epidemiology (MOOSE) guideline.^38^

### Data Search and Extraction Strategy

**Figure 1.**
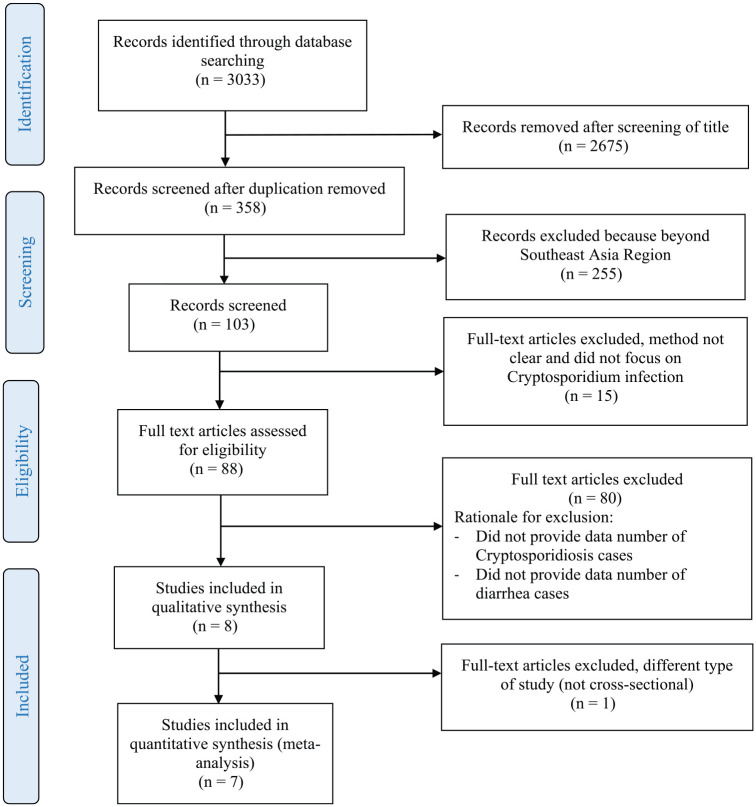
Flowchart describing the study design process.

The methodology used was based on the Preferred Reporting Items for Systematic Reviews and Meta-Analyses (PRISMA). Literature was collected using PubMed, Science Direct, ProQuest, EBSCO, Cochrane, and Web of Science databases. The keywords were “*Cryptosporidium*,” “cryptosporidiosis,” cross-referenced with “HIV,” “immunodeficiency,” “acquired immune deficiency syndrome,” or “AIDS,” without language restriction. The literature search was narrowed down to Southeast Asia region, using following keywords: Indonesia, Malaysia, Singapore, Thailand, Myanmar, Laos, the Philippines, Brunei, Vietnam, and Cambodia. Research subjects were defined as a research with human subjects, written in English, peer-reviewed, and available in full text. Using these inclusion criteria, there were 88 selected citations. Articles that did not display the number of PLHIV who suffered from chronic diarrhea or persistent diarrhea were excluded. Furthermore, studies that did not provide a prevalence estimation or any sufficient information from which a prevalence could be calculated were excluded. Time of publication was limited from January 2005 to December 2017 because meta-analysis research on cryptosporidiosis was previously done in 2004.^39^ Based on this, a literature study was conducted from 2005 to the end of 2017. The article selection process is shown in Figure 1.

### Data Analysis

To combine the results of various studies is the most decisive part of a meta-analysis. A higher quality of research usually has a greater weight in meta-analysis. The heterogeneity of the effect size was tested using the Cochrane *Q* test and *I*^2^ statistics. Statistical heterogeneity values of 25%, 50%, and 75% reflect low, medium, and high heterogeneity values, respectively. If the value of heterogeneity through the Cochrane *Q* test was significant or *I*^2^ ≥25%, the random effect size method was used to estimate the pooled effect size, in contrast to if the heterogeneity was <25%.^40^ The *I*^2^ value >50% represent substantial heterogeneity, while the *I*^2^ value >75% represent high heterogeneity between the trials in this study. Relative risk (RR) was used to determine the effect size of each study variable and its relationship to the risk of chronic diarrhea in *Cryptosporidium* infection. Publication bias among all studies was tested using a funnel plot. The analysis was performed by STATA version 13.0 statistical software (Stata Corp, College Station, TX).

## Results

This study obtained 7 available studies for the meta-analysis (n = 854) as shown in Tables 1 and 2. The effect size of each study resulted in RR >1, was varying from 1.239 to 3.556, which represented variable of *Cryptosporidium* infection was a risk factor for chronic diarrhea among PLHIV. Two studies showed a good effect size, 1.24^41^ and 1.37,^42^ respectively, as shown in Figure 2 (see supplemental file, available online). The effect size was traversed by a dotted line that crosses the diagonal of the diamond with a narrow confidence interval (CI). Furthermore, only one study^18^ reported a protective effect, which *Cryptosporidium* infection does not necessarily cause chronic diarrhea in HIV-seropositive patients.

**Table 1.** Characteristics of Included Studies.

S. No.	Reference	Location	Type of Study	Study Duration	Number of HIV Cases	Number of Cryptosporidiosis Cases	Number of Diarrhea Cases
1	Idris et al^48^	Jakarta, Indonesia	Cross-sectional	April 2008 to February 2009	42 cases	2 cases	15 cases
2	Saksirisampant et al^41^	Thailand	Cross-sectional	During 2005	90 cases	31 cases	71 cases
3	Kurniawan et al^18^	Jakarta, Indonesia	Cross-sectional	November 2004 to March 2007	318 cases	13 cases	263 cases
4	Srisuphanunt et al^42^	Thailand	Cross-sectional	Not available	143 cases	23 cases	101 cases
5	Pinlaor et al, 2005^71^	Thailand	Cross-sectional	November 1998 to August 2000	78 cases	9 cases	25 cases
6	Paboriboune et al^56^	Laos	Cross-sectional	October 2009 to September 2010	137 cases	9 cases	20 cases
7	Nuchjangreed et al^51^	Thailand	Cross-sectional	January to August 2007	46 cases	13 cases	13 cases

**Table 2.** Systematic Review of Included Studies.

S. No.	Author	Title	Prevalence	Detection Methods	Findings
1	Srisuphanunt et al^42^	Potential risk factors for *Cryptosporidium* infection among HIV/AIDS patients in central areas of Thailand	16.1%	Modified Acid-Fast staining (MAF staining)	*Cryptosporidium* infection detected with a history of diarrhea >21 days (OR = 2.8, 95% CI = 1.072-7.283, *P* = .031), CD4^+^ count ≤50 cells/mm^3^ (OR = 11, 95% CI = 1.387-87.986, *P* = .006).
2	Nuchjangreed et al^51^	Prevalence and molecular characterization of human and bovine *Cryptosporidium* isolates in Thailand	28.7% by AFS and 4.35% by nested PCR	MAF staining and nested PCR 18S rRNA	There was no significant difference between the number of patients who were positive for *Cryptosporidium* and diarrhea with the number of positive patients with *Cryptosporidium* without diarrhea (*P* > .05).
3	Saksirisampant et al^41^	Intestinal parasitic infections: prevalences in HIV/AIDS patients in a Thai AIDS care center	23.3% by microscopy, 36.7% by PCR	MAF staining and nested PCR 18S rRNA	The consistency of abnormal stools (mucoid, loose-watery or watery consistency) of patients can help in the initial diagnosis of opportunistic protozoan infections and allow further parasitological investigations that may be useful in the initial screening for these parasitic infections in HIV/AIDS cases.
4	Pinlaor et al, 2005^71^	Detection of opportunistic and nonopportunistic intestinal parasites and liver flukes in HIV-positive and HIV-negative subjects	11.5%	MAF staining and direct fluorescence techniques	It is necessary to periodically check for possible reemergence of infectious organisms. Need to raise awareness of rural communities about the education program about AIDS.
5	Paboriboune et al^56^	Intestinal parasitic infections in HIV-infected patients, Lao People’s Democratic Republic	6.6%	MAF staining	Immunocompromised patients with CD4 counts <50 are more likely to be infected with *Cryptosporidium* than CD4 cells> 200 and tend to be diagnosed at an advanced stage.
6	Kurniawan et al^18^	Intestinal parasitic infections in HIV/AIDS patients presenting with diarrhea in Jakarta, Indonesia	4.9%	MAF staining	Recommendations for routine examination of intestinal parasites in people with HIV/AIDS, especially those whose immunity decreases or the number of CD4^+^ cells is low because low immunity predisposes to intestinal parasites and the onset of prolonged diarrhea for more than 4 weeks.
7	Idris et al^48^	Intestinal parasitic infection of immune-compromised children with diarrhea: clinical profile and therapeutic response	9.1%	MAF staining	The study also recommends routine examination of intestinal parasites in the feces of immunocompromised children with persistent and or recurrent diarrhea. Children and toddlers are the main groups affected by cryptosporidiosis, especially children infected with HIV who are not taking antiretroviral therapy.

Abbreviations: OR, odds ratio; CI, confidence interval; AFS, acid-fast stain; PCR, polymerase chain reaction.

The fixed effect size model using pooled effect size of the 7 studies obtained RR of 1.325 (*P* = .000, 95% CI = 1.157-1.517), which is statistically significant, and representing the risk of chronic or persistent diarrhea was 1.325 times higher in PLHIV. Substantial heterogeneity or *I*^2^ was 72% (*P* = .002), indicating that variations between studies differ significantly. This finding might be because the magnitudes of the intervention effects were varied greatly between and intra-studies; therefore, if the analyses are performed on different populations, times, places, and conditions, the results will be different.

Heterogeneity in the meta-analysis leads to variations in each research outcome among several of these studies. The standard measurement of heterogeneity is Cochrane’s *Q*, which is calculated as the sum of weights from the square of the difference between the individual effect size and the combined effect size of all studies. *Q* has a low power as a comprehensive test for heterogeneity when the number of study is few. Inversely, *Q* has a high power as a heterogeneity test if the number of study is significant enough.^43^

All the studies have a weighted average value that varies from 1.41 to 33.78. Three studies have a good weighted average, Saksirisampant et al^41^ (33.78%), Srisuphanunt et al^42^ (29.35%), and Kurniawan et al^18^ (23.17%). A funnel plot graph was used to illustrate publication bias. The results were considered to be statistically significant if the value was *P* < .05. The description of the effect size of the 7 studies is shown in Figure 2 (see supplemental file, available online).

The relationship between CD4^+^ T-lymphocyte cell count and opportunistic infections such as *Cryptosporidium* has been widely reported.^42,44,45^ The number of T-lymphocyte ≤100 cells/mm^3^ or between 101 and 200 cells/mm^3^ indicates a high risk of parasitic infection, while cryptosporidiosis was also reported to be closely related to low CD4^+^ counts in several studies.

We conducted the meta-analysis of the 7 selected studies to review the relationship between exposure to *Cryptosporidium* infection and the amount of CD4^+^ T-lymphocyte in PLHIV. There were only three out of seven studies used to carry out further meta-analysis (n = 507). The results of the meta-analysis on the dependent variable of cryptosporidiosis were related to the independent variable number of CD4^+^ T-lymphocyte. Pooled effect size of this variable with RR = 1.206 (95% CI = 0.911-1.598), *P* = 0.191, indicated that there was no significant relationship between cryptosporidiosis and the number of CD4^+^ T-lymphocyte. The value of substantial heterogeneity *I*^2^ was 0.0% (*P* = .442), suggesting variations between the studies were homogeneous but not significant. Since the RR values were above 1 and below 2, which is empirically classified as low, this accounted for 0.0% in heterogeneity. Only one out of three studies showed significant RR values between 1 and 2. The relationship between the two variables is shown in the forest plot in Figure 3 (see supplemental file, available online).

## Discussion

Diarrhea is defined as the passage of three or more loose or liquid stools per day (or more frequent passage than is normal for the individual). It is usually a symptom of gastrointestinal infection caused by a variety of bacterial, viral, and parasitic organisms, which may spread through contaminated food or drinking water or from person to person as a result of poor hygiene.^46^ According to the World Health Organization, classic diarrhea is generally differentiated into acute and chronic based on its duration. Acute diarrhea is described as having acute onset and duration of not more than 14 days, whereas chronic or persistent diarrhea is defined as having an onset of more than 14 days. Prolonged diarrhea often has a series of different causes that requires different management and shows different prognosis.^47^

We conducted a meta-analysis of several studies on human cryptosporidiosis involving HIV-seropositive subjects in Southeast Asia countries. Only 3 out of 11 Southeast Asia countries were included: Indonesia, Thailand, and Laos.

Human cryptosporidiosis is known as an intestinal protozoan infection with different clinical characteristics between immunocompetent and immunosuppressed individuals. Although it is self-limiting in immunocompetent people, it can be a potential life-threatening infection for those with immune defects, especially HIV. This research was similar to a study conducted in Jakarta, Indonesia, which showed 10 out of 474 children (2.1%) were positive for cryptosporidiosis, while all suffered from malnutrition and 40% of them had a chronic diarrhea. Another study conducted by Idris et al^48^ on children aged 1 to 5 years with immunocompromised status (HIV, malignancy, or other causes) showed a prevalence of 9.1% cryptosporidiosis in HIV-seropositive children. This results were lower than other findings conducted by Kurniawan et al,^18^ which showed a prevalence of 11.9% cryptosporidiosis in HIV-seropositive children in Jakarta and a prevalence of 12.8% cryptosporidiosis in HIV-seropositive children in Thailand. These findings indicated that the presence of *Cryptosporidium* infection can be a marker of severe immune deficiency and were associated with very low CD4^+^ counts in PLHIV, especially in children. These studies showed that children with chronic diarrhea or recurrent diarrhea tend to have parasitic infections such as *Cryptosporidium* 1.8 times more often than those without diarrhea.^48^

Rashmi and Kumar^49^ suggested a correlation between *Cryptosporidium* infection in HIV-seropositive patients and their CD4^+^ cell count. Patients with CD4^+^ <100 cell/mm^3^ have a higher risk of *Cryptosporidium* infection than those with CD4^+^ >100 cell/mm^3^. The characteristics of diarrhea and other symptoms differ between HIV-seronegative and HIV-seropositive people, as well as HIV-seropositive people with different CD4^+^ counts. In general, symptoms are more severe in HIV-positive patients, especially for those with CD4^+^ <100 cell/mm^3^. Asymptomatic infections of cryptosporidiosis are characterized by unchanging bowel habits less than 3 times a day but positive laboratory examination of feces. This temporary infection generally lasts less than 2 months and is associated with an average CD4^+^ count above 200 cells/mm^3^ and loss of oocysts from feces. Diarrhea resolves typically without any use of antidiarrheal drugs, and the common infection is 36 weeks. Failure diagnosing cryptosporidiosis in immunocompetent patients with diarrhea often occurs, although it is infrequent since it is self-limited disease. However, it is totally different in immunocompromised patients, due to its severity and treatment procedures.^49^ Cryptosporidiosis in PLHIV and other immunocompromised patients tends to last longer and can be chronically progressive in susceptible individuals, such as children with malnutrition.^50^

Srisuphanunt et al^42^ showed that 17.4% of 143 HIV-seropositive patients in Thailand suffered from chronic and persistent diarrhea, in which 69.5% were positive for *Cryptosporidium*, compared with 9.2% and 45% in negative infection. The effect size was generated from the meta-analysis (RR = 1.370, 95% CI = 1.146-1.637), and its weight was 29.35%, indicating that this study significantly supports the role of *Cryptosporidium* infection in chronic diarrhea in HIV-seropositive patients. Moreover, Saksirisampant et al^41^ reported a similar meta-analysis with a good effect size (RR = 1.239, 95% CI = 1.021-1.504), in which 31 out of 90 (36.7%) HIV-seropositive patients were infected with *Cryptosporidium*, and 28 patients (90.32%) showed clinical symptoms of chronic diarrhea.

Different laboratory examination techniques for detecting *Cryptosporidium* infection may also influence the value of heterogeneity of the studies. All studies performed modified acid-fast (MAF) staining examination for detection of *Cryptosporidium* infection. Moreover, several studies conducted additional confirmation tests, such as polymerase chain reaction (nested PCR method) and direct fluorescence techniques. In the majority of studies, recent confirmation tests such as PCR have a better quality because they can detect infections that were previously declared negative by a conventional examination. But not all studies stated their findings, because false-positive results could happen from conventional examination, as reported by Nuchjangreed et al.^51^ Two out of seven studies showed good experimental design, which were conducted by Srisuphanunt et al,^42^ performed MAF staining as a detection methods, while Saksirisampant et al^41^ performed microscopic techniques followed by nested PCR, which was more sensitive than the staining method alone. The other study conducted by Nuchjangreed et al^51^ revealed 28.7% and 4.35% *Cryptosporidium* infection in PLHIV (with and without chronic diarrhea) by microscopic examination and PCR method. The difference of cryptosporidiosis between those with and without diarrhea was not significant (*P* > .05), indicating that *Cryptosporidium* infection is not always symptomatic even in PLHIV. These results are in line with other studies that suggested asymptomatic cryptosporidiosis in PLHIV,^44,52,53^ with various incidence rates, such as in 8% to 32% in Korea^54^ and 16.7% in Tanzania.^55^ However, in general, *Cryptosporidium* infection is more often accompanied with diarrhea.

Paboriboune et al^56^ suggested that 83.9% of their study population were severely immunocompromised (at World Health Organization stage 3 or 4) with CD4^+^ cell counts <50 cells mm^3^. According to their study, the majority of PLHIV in Laos visit the medical office in the late stage of disease due to three conditions: (1) majority of PLHIV (54%) live in villages with few or no access to information about the harmful effects of AIDS and its prevention, (2) HIV screening services are not available in the nearest health care services, and (3) people often use traditional medicine and only seek for medical treatment if their health condition has been deteriorated.^56^

The etiological diagnosis of cryptosporidiosis can be performed by microscopic diagnosis methods, antigen detection with immunoassay, and molecular diagnosis approaches. A large number of oocysts (at least 1 × 10^6^/mL) is needed for microscopic examination. Moreover, well-trained and experienced laboratory officers will be needed, and the examination process requires a longer time.^57^ At early stage of infection when the oocysts have not been released in large quantities in the feces, the microscopic examination tends to be negative. Furthermore, the oocysts will be released intermittently with varied amount day by day. In cases with high tendency of cryptosporidiosis but no oocysts can be found in feces, it is necessary to confirm with other techniques such as antigen detection by ELISA (enzyme-linked immunosorbent assay) or other advanced examination.^58,59^ Detection of *Cryptosporidium* oocysts from pulses stained by acid-resistant modification methods show a high specificity with low sensitivity; thus, it becomes challenging to detect asymptomatic cases or low-intensity parasitic infections.^60^ Therefore, it is necessary to diagnose cryptosporidiosis using another detection technique other than microscopic examination.

Since the microscopic detection methods have a low sensitivity and are more difficult to obtain accurate results, the application of certain molecular technology is critical to obtain epidemiological data of cryptosporidiosis and genotypes of *Cryptosporidium*, to support prevention and control strategies.^61^ Currently, there are many more molecular examination methods being developed, especially for identifying *Cryptosporidium* species and evaluating their treatment.^62-64^

The molecular characterization of the *Cryptosporidium* species and genotyping can also accurately prove the existence of zoonotic transmission in the epidemiology of cryptosporidiosis.^65^ Kurniawan et al^15^ confirmed that a significant difference between the routine examination of *Cryptosporidium* and MAF staining was 4.8% and PCR obtained 34.6% using the 18S rRNA gene. The results showed the actual high prevalence of *Cryptosporidium* infection, even when most of them were asymptomatic. The use of PCR technique to detect *Cryptosporidium* infection is beneficial especially when dealing with many specimens or when encountering cases with very few oocysts. While less sensitive for mass diagnosis in public services in hospitals and health laboratories, MAF staining (as a gold standard) is beneficial for public services in hospitals and health laboratories, where there are not too many specimens, while it is less sensitive for mass diagnosis. Current PCR procedures have been evaluated and developed to examine genotypes and specific *Cryptosporidium*, while cell cultures and animal models are used to evaluate chemotherapy and immunotherapy agents.^66^

The correlation between intestinal protozoa infection, in this case, *Cryptosporidium*, as an opportunistic parasite and the decreasing of immunity characterized by depletion of CD4^+^ T-lymphocyte cells in PLHIV has been proven.^18,44^ An Ethiopian study reported that parasitic infections were more accessible to infect PLHIV than non-HIV persons, and the cohort study showed that number of CD4^+^ T-lymphocyte cells <50 cell/mm^3^ was more commonly found in those who were infected by parasites.^67^ In Jakarta, other studies showed that 74% of HIV-seropositive patients with diarrhea more than 4 weeks had CD4^+^ T-lymphocyte <100 cell/mm^3^, and their clinical condition was even worse in patients with CD4^+^ T-lymphocyte cell counts <50 cell/mm^3^. The severity of diarrhea and duration of clinical symptoms are associated with CD4^+^ T-lymphocyte counts. The risk of clinical symptoms are increased along with the decreasing of CD4^+^ T-lymphocyte; therefore, people with CD4^+^ cell counts between 100 and 199 cell/mm^3^ possess a more severe risk of disease compared with people with CD4^+^ T-lymphocyte >200 cells/mm^3^. Individuals with low CD4^+^ T-lymphocyte cell counts increase the risk of parasitic intestinal infections being opportunistic agents.^42^ Simultaneous activation of CD4^+^ T-lymphocyte cells and interferon-γ (IFN-γ) is required to prevent *Cryptosporidium* infection.^49^ CD4^+^ T-lymphocyte cells are useful for limiting the duration of disease, while IFN-γ serves to limit the intensity of the infection. The increasing risk of contracting the infection from infected contacts and prolonged excretion of *Cryptosporidium* correlates with the high prevalence of this disease in PLHIV.^42^

Antiretroviral therapy is still one of the therapeutic interventions that showed a remarkable effect on cryptosporidiosis in HIV-seropositive patients because it leads to the recovery of CD4^+^ counts. ART can reduce the frequency and severity of cryptosporidiosis in PLHIV.^68^ A correlation study between *Cryptosporidium* infection with the CD4^+^ counts of patients in India showed that the HIV-seropositive patients with CD4^+^ <100 cells/mm^3^ were 6.09 times more susceptible to be infected by *Cryptosporidium* (*P* = .002).^49^ The findings were consistent with other reports by Sadraei et al^69^ and Wiwanitkit,^44^ which reported *Cryptosporidium* as an opportunistic infection in HIV-seropositive patients with CD4^+^ <200/µL. Other research study by Paboriboune et al^56^ showed that a relatively good effect size with RR = 1.422 (95% CI = 1.089-1.858) and a weight of 31.62% indicated the risk of cryptosporidiosis against low CD4^+^ T-lymphocyte counts. Kurniawan et al^18^ also reported similar findings even though with smaller effect sizes, RR = 1.126 (95% CI = 0.720-1.759) and weight of 40.31%, as well as RR = 1.079 (95% CI = 0.506-2.302) and weight of 28.06% reported by Srisuphanunt et al,^42^ although the pooled effect size was not significant, wherein low heterogeneity might be due to few samples, which tend to be homogeneous. Since *Cryptosporidium* infection is related to the risk of chronic diarrhea, the clinicians must pay attention to the number of CD4^+^ T-lymphocyte cells; the lower the CD4^+^ cells counts, the greater the risk of chronic diarrhea with prolonged duration.^13^ Previous studies suggested that additional laboratory examinations must be conducted when diagnosing a person as HIV-seropositive.

In immunocompetent hosts, restoration of immune function is a key component of patient management. Immune reconstitution in response to an effective combination of ART has been related to parasite clearance, as well as reduced long-term morbidity and mortality associated with *Cryptosporidium* infection of patients with AIDS. Symptomatic therapy is indispensable in cryptosporidiosis. Fluid and electrolyte replacement was preferred as in other causes of diarrhea. Some drugs, such as paromomycin, may reduce the symptoms of cryptosporidiosis.^70^ Antimotility drugs can be given as adjuvant therapy. Although the administration of ART is quite adequate, chronic diarrhea in PLHIV is associated with an early mortality. To date, an established curative therapy is not yet available for cryptosporidiosis.^11^ Currently, there is no vaccine available for preventive therapy. Moreover, the only drug approved by US Food and Drug Administration for cryptosporidiosis, nitazoxanide, is not effective in immunocompromised hosts.^17^ One research showed that nitazoxanide has a killing effect on parasites in non-HIV patients.^16^ Aforementioned findings suggested that we should find an active therapeutic agent for *Cryptosporidium* as a research priority.

## Conclusion

The pooled size effect of all studies showed a statistically significant relationship between the risk of chronic diarrhea and cryptosporidiosis in PLHIV. This result suggested that cryptosporidiosis increases the risk of chronic diarrhea, and low CD4^+^ T-lymphocyte cell counts can aggravate the degree of diarrhea. Practitioners should pay attention on clinical and paraclinical characteristics of the PLHIV in diagnosing cryptosporidiosis, and other examination for the detection of the opportunistic intestinal protozoan infection should use clinical and paraclinical characteristics of the PLHIV for the diagnosis of *Cryptosporidium* and other opportunistic parasitic diseases in clinical management.

## Supplemental Material

Figure_2._The_pooled_relative_risk_RR_between_cryptosporidiosis_and_chronic_diarrhea – Supplemental material for Cryptosporidium Infection Increases the Risk for Chronic Diarrhea Among People Living With HIV in Southeast Asia: A Systematic Review and Meta-Analysis Click here for additional data file. Supplemental material, Figure_2._The_pooled_relative_risk_RR_between_cryptosporidiosis_and_chronic_diarrhea for Cryptosporidium Infection Increases the Risk for Chronic Diarrhea Among People Living With HIV in Southeast Asia: A Systematic Review and Meta-Analysis by Wiwien S. Utami, Elsa H. Murhandarwati, Wayan T. Artama and Hari Kusnanto in Asia Pacific Journal of Public Health

Figure_3._The_pooled_relative_risk_RR_between_cryptosporidiosis_related_to_CD4plus_lymphocyte_counts. – Supplemental material for Cryptosporidium Infection Increases the Risk for Chronic Diarrhea Among People Living With HIV in Southeast Asia: A Systematic Review and Meta-Analysis Click here for additional data file. Supplemental material, Figure_3._The_pooled_relative_risk_RR_between_cryptosporidiosis_related_to_CD4plus_lymphocyte_counts. for Cryptosporidium Infection Increases the Risk for Chronic Diarrhea Among People Living With HIV in Southeast Asia: A Systematic Review and Meta-Analysis by Wiwien S. Utami, Elsa H. Murhandarwati, Wayan T. Artama and Hari Kusnanto in Asia Pacific Journal of Public Health
