# Supplementary material for: Cryptosporidium Infection Increases the Risk for Chronic Diarrhea Among People Living With HIV in Southeast Asia: A Systematic Review and Meta-Analysis
Source: Asia Pac J Public Health. 2020 Feb 10;32(1):8–18. doi: 10.1177/1010539519895422 (PMC7750677; doi:10.1177/1010539519895422)
Supplement: Figure_3._The_pooled_relative_risk_RR_between_cryptosporidiosis_related_to_CD4plus_lymphocyte_counts. – Supplemental material for Cryptosporidium Infection Increases the Risk for Chronic Diarrhea Among People Living With HIV in Southeast Asia: A Systematic Review and Meta-Analysis [file Figure_3._The_pooled_relative_risk_RR_between_cryptosporidiosis_related_to_CD4plus_lymphocyte_counts..pdf]

Figure 3. The pooled relative risk (RR) between cryptosporidiosis related to CD4$^+$ lymphocyte counts.

Supplemental material for *Cryptosporidium* Infection Increases the Risk for Chronic Diarrhea among People Living with HIV in Southeast Asia: A Systematic Review and Meta-Analysis.

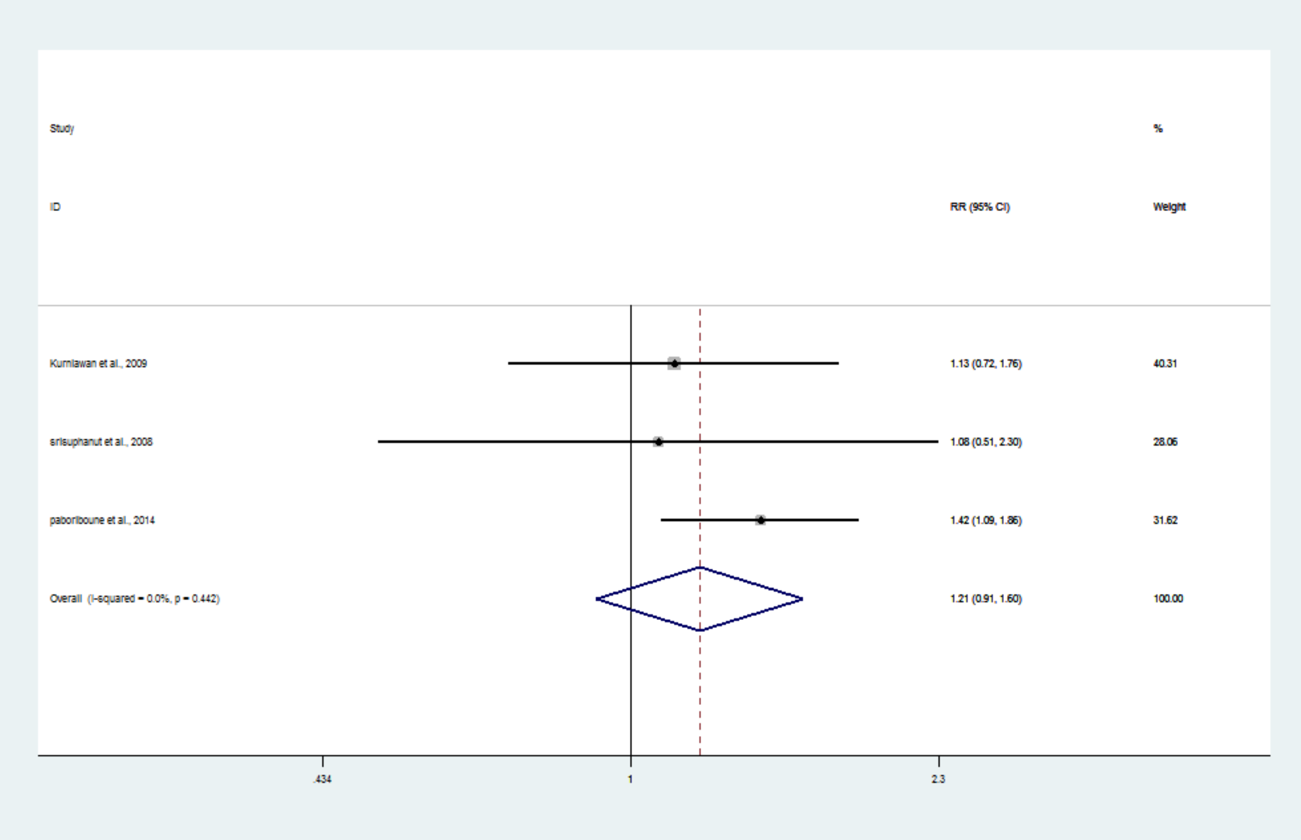

Abbreviations: CI, confidence interval, RR, relative risk.

Figure 3. The pooled relative risk (RR) between cryptosporidiosis related to CD4$^+$ lymphocyte counts.
